# Hepatitis B Virus X Protein Inhibits the Expression of Barrier To Autointegration factor1 via Upregulating miR-203 Expression in Hepatic Cells

**DOI:** 10.1128/spectrum.01235-22

**Published:** 2022-12-15

**Authors:** Amit Kumar Mishra, Md Musa Hossain, Teja Naveen Sata, Ajay K. Yadav, Shahidullah Zadran, Amrendra Kumar Sah, Baibaswata Nayak, Shalimar, Senthil Kumar Venugopal

**Affiliations:** a Faculty of Life Sciences and Biotechnology, South Asian University, Chanakyapuri, New Delhi, India; b All India Institute of Medical Sciences (AIIMS), New Delhi, India; Indian Institute of Science Bangalore

**Keywords:** BANF1, HBx, hepatitis B virus, host restriction factor, microRNA-203

## Abstract

Hepatitis B virus (HBV) infection targets host restriction factors that inhibit its replication and survival. Previous studies have shown that barriers to autointegration factor1 (BANF1) inhibited the replication of herpes simplex virus and vaccinia virus by binding to phosphate backbone of dsDNA. To date, no reports are available for the interplay between BANF1 and HBV. In this study, we elucidated the mechanisms by which HBV inhibit BANF1. First, the effect of HBV on BANF1 was observed in Huh-7, Hep G2, and Hep G2.2.15 cells. Huh-7 cells were transfected with pHBV_1.3_ or HBx plasmids. The results showed that there was a decreased expression of BANF1 in Hep G2.2.15 cells (*P* ≤ 0.005) or in HBV/HBx expressing Huh-7 cells (*P* ≤ 0.005), whereas BANF1 overexpression decreased viral replication (*P* ≤ 0.05). To study whether phosphorylation/dephosphorylation of BANF1 was responsible for antiviral activity, mutants were created, and it was found that inhibition due to mutants was less significant compared to BANF1 wild type. Previous studies have shown that HBV, at least in part, could regulate the expression of host miRNAs via HBx. It was found that miR-203 expression was high in Hep G2.2.15 cells (*P* ≤ 0.005) compared to Hep G2 cells. Next, the effect of HBx on miR-203 expression was studied and result showed that HBx upregulated miR-203 expression (*P* ≤ 0.005). Overexpression of miR-203 downregulated BANF1 expression (*P* ≤ 0.05) and viral titer was upregulated (*P* ≤ 0.05), while inhibition of miR-203, reversed these changes. In conclusion, BANF1 downregulated HBV, whereas HBV inhibited BANF1, at least in part, via HBx-mediated miR-203 upregulation in hepatic cells.

**IMPORTANCE** In this study, for the first time, we found that BANF1 inhibited HBV replication and restricted the viral load. However, as previously reported for other viruses, the results in this study showed that BAF1 phosphorylation/dephosphorylation is not involved in its antiviral activity against HBV. HBV infection inhibited the intracellular expression of BANF1, via HBx-mediated upregulation of miR-203 expression. Overexpression of miR-203 downregulated BANF1 and increased the viral titer, while inhibition of miR-203 reversed these changes. This study helped us to understand the molecular mechanisms by which HBV survives and replicates in the host cells.

## INTRODUCTION

More than 350 million people are suffering from chronic hepatitis B virus (HBV) infection and it is a global public health problem. Individuals suffering from chronic hepatitis B infection, carry a high risk for developing liver cirrhosis and hepatocellular carcinoma (HCC) (1). Upon entry into the host cells, HBV interacts with numerous cellular factors, such as, host immune system, miRNAs and other cellular proteins, and modulate their expression to survive in the host cells. Recently, SMC5/6 has been characterized to possess potential antiviral activity against HBV (2). Talin1 is another protein of the same series, characterized to inhibit HBV replication (3). In both the studies, HBV encoded HBx protein was reported to be requisite to rescue HBV inhibition. HBV encodes HBx protein, surface antigen HBsAg, HBV core protein HBcAg and HBV polymerase (Pol) which is basically reverse transcriptase. Several studies have shown that HBx is indispensable for HBV replication. HBx acts directly on viral promoters and enhancers to promote viral replication (4). HBx indirectly also contributes to viral replication by modulating cellular pathways to benefit HBV infection (2, 5). Apart from host proteins, various miRNAs have been shown to control HBV replication, including the epigenetic modulation of HBV genome (6). Recent studies show that HBV itself encodes a miRNA (HBV-miR-3) which restricts HBV replication by activating the innate immune system (7, 8). HBV accessory protein HBx also modulates the expression of various miRNAs that are involved in innate and adaptive immune pathways, and thus, helps in HBV replication and survival (9). BANF1 is a small (89 residues; 10 kD) protein present in the nucleus of cells and is capable of forming stable homodimers that can bind two dsDNA molecules (10). Each monomer of BANF1 consists of two copies of the helix-hairpin-helix (HhH) motif that can bind dsDNA in a sequence independent manner (11). BANF1 binds the phosphate backbone of dsDNA, and thus, lacks or does not require DNA sequence specificity (10, 11). Originally BANF1 was identified in mammalian cells by virtue of its association with the preintegration complexes (PICs) of retroviruses (12). BANF1 acts as a potent antiviral effector against the poxvirus vaccinia virus in the cytoplasm due to its DNA-binding/compaction activity. Poxviruses express a Ser/Thr protein kinase named B1 kinase which phosphorylates BANF-1 at its N-terminal; this in turn inactivates its DNA binding capability (13). BANF1 is a threat not only to cytoplasmic poxviruses but for nuclear DNA viruses as well. BANF1 has been reported to interfere with herpes simplex virus 1 (HSV-1) viral DNA replication and gene expression leading to viral inhibition (14). Over the past decade, many efforts have been made into identification of HBV interacting host cell proteins. To date, several host dependency factors also known as proviral factors have been identified that are beneficial for HBV and act positively to support HBV replication. This puts host cells under tremendous selective pressure to prevent HBV infection, thus leading toward evolution of various restriction factors that act negatively to inhibit or block HBV. Because BANF1 can crossbridge DNA and induces DNA condensation by cross-linking different regions of DNA, we hypothesized that BANF1 might restrict the HBV, and hence, HBV might inhibit the expression of BANF1.

## RESULTS

### HBV infection downregulates BANF1 expression.

**FIG 1:**
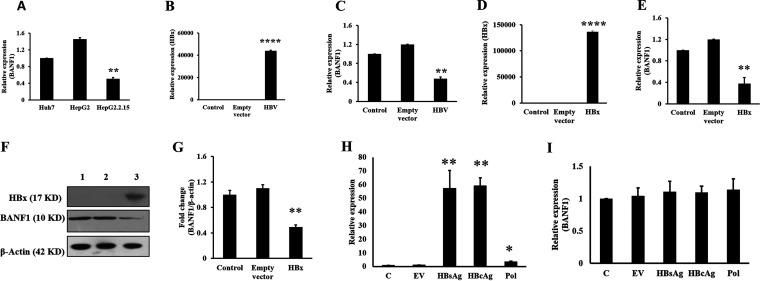
HBx downregulates BANF1 expression in hepatoma cells. Huh-7, Hep G2, and Hep G2.2.15 cells were cultured, and the total RNA was isolated followed by RT-PCR for BANF1 expression (*n* = 3; **, *P* < 0.01) (A). Huh-7 cells were transfected with pHBV_1.3_ plasmid and the cells were collected for the isolation of RNA, followed by RT-PCR for HBx (B) or BANF1 (C) expression (*n* = 3; ***, *P* < 0.001; **, *P* < 0.01). Huh-7 cells were transfected with HBsAg, HBcAg and HBV-pol plasmid, total RNA was isolated and RT-PCR was performed for HBsAg, HBcAg and HBV-pol (*n* = 3; *, *P* < 0.05; **, *P* < 0.01) (H) and BANF1 (I). After 48 h, both the RNA and protein were isolated and RT-PCR or Western blots were performed. The expression of HBx (D) or BANF1 (E) was determined (*n* = 3; ***, *P* < 0.001; **, *P* < 0.01). The total cellular protein was run on SDS-PAGE gels and Western blots were performed and a representative picture is shown. Lane 1, control; lane 2, Empty vector transfected cells; and lane 3, HBx-transfected cells (F). The band intensities were quantified using Image J software and presented as fold change (BANF1/β-actin) *n* = 3; **, *P* < 0.01 (G).

Effects of HBV infection on BANF1 expression was analyzed by real-time PCR (RT-PCR) in Huh-7 cells Hep G2 cells and HBV expressing Hep G2.2.15 cells. The results showed that BANF1 expression was decreased by 2-fold in HBV expressing Hep G2.2.15 cells in comparison to Huh-7 cells and Hep G2 cells (Fig. 1A). Further, Huh-7 cells were transfected with HBV plasmid for 48 h and total RNA was isolated. RT-PCR results revealed that there was a significant increase in HBx expression (Fig. 1B), while BANF1 expression was decreased by 60 percent in HBV-transfected Huh-7 cells (Fig. 1C). HBx is requisite to initiate and maintain HBV replication. To study whether HBx played a role in the BANF1 expression, Huh-7 cells were transfected with HBx plasmid. After 48 h of transfection, the cells were harvested for RNA and total protein isolation. RT-PCR results showed significant increase in HBx expression in the HBx-transfected cells (Fig. 1D). BANF1 expression was significantly downregulated in HBx-transfected Huh-7 cells (Fig. 1E). Western blot results showed that there was significant decrease in BANF1 expression in HBx transfected Huh-7 cells (Fig. 1F). The BANF1 band intensity quantification showed that BANF1 was downregulated by 65% in HBx-transfected cells (Fig. 1G). To further validate these results, Huh7 cells were transfected with HBsAg, HBcAg, and HBV polymerase (three additional proteins encoded by HBV apart from HBx) expressing plasmids, and performed RT-PCR to evaluate the change in expression of BANF1. Results showed that HBsAg, HBcAg and HBV-pol were upregulated (Fig. 1H) but there was no change in expression of BANF1 in HBsAg, HBcAg, and HBV pol transfected cells (Fig. 1I). Hence, it was concluded that the decrease in BANF1 expression was due to HBx, but not due to HBsAg, HBcAg and HBV pol. These results suggest that HBV infection downregulated BANF1 expression via HBx protein.

### BANF1 overexpression inhibits HBx, HBsAg, and the viral load.

**FIG 2:**
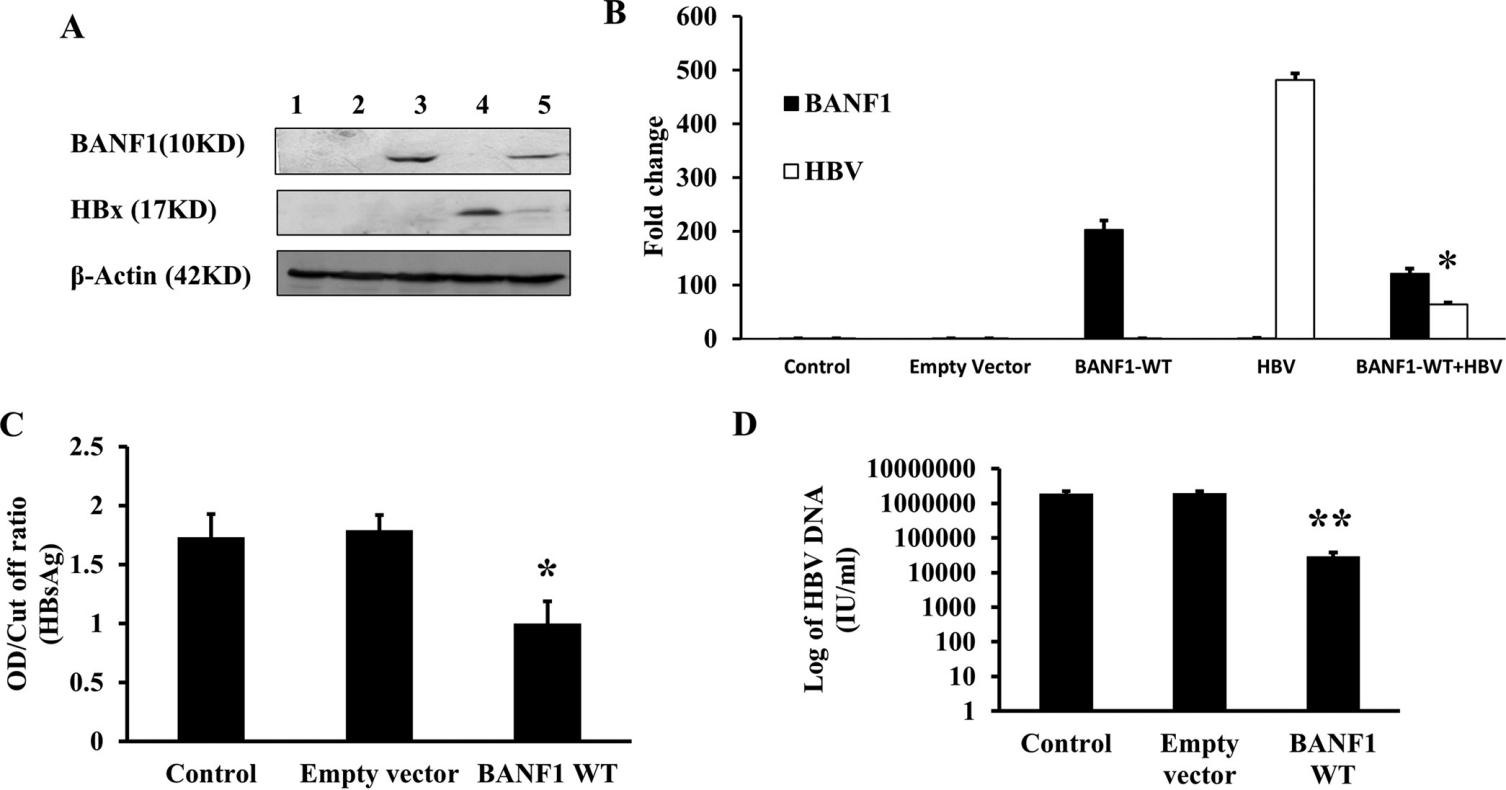
BANF1 overexpression inhibits expression of HBx, HBsAg and HBV replication. Huh-7 cells were transfected either alone with BANF1 plasmid, pHBV_1.3_ plasmid or cotransfected together. After 48 h, cells were harvested for total cellular protein isolation and Western blots were performed. A representative picture is shown. Lane 1, control; lane 2, empty vector transfected cells; lane 3, BANF1 transfected cells; lane 4, pHBV_1.3_ transfected cells; lane 5, BANF1 and pHBV_1.3_ cotransfected cells (A). The band intensities were quantified using Image J software and presented as fold change (*n* = 3; *, *P* < 0.05) (B). Hep G2.2.15 cells were transfected with BANF1 (WT) plasmid and after 48 h media was collected for HBsAg analysis by Enzyme Linked Fluorescent Assay (ELFA) (*n* = 3; *, *P* < 0.05) (C) and cells were harvested for HBV DNA isolation and quantification by qPCR (*n* = 3; ***, *P* < 0.001) (D).

In order to investigate the effect of BANF1 on HBV life cycle, BANF1 gene was amplified from Huh-7 cells, cloned into pcDNA3.1(-) vector and confirmed by sequencing. BANF1 plasmid was transfected either alone or cotransfected with HBV_1.3_ plasmid (pHBV_1.3_) in Huh-7 cells. Western blot results showed that BANF1 overexpression downregulated HBx expression significantly (Fig. 2A). BANF1 and HBx band intensity quantification showed that HBx expression was downregulated in BANF1-overexpressing cells (Fig. 2B). Furthermore, Hep G2.2.15 cells were transfected with BANF1 plasmid and cultured for 48 h. Then, the spent media and the cells were collected for HBsAg analysis by ELISA and HBV DNA analysis by real-time PCR, respectively. HBsAg titer was significantly low in BANF1-transfected cells compared to the control or empty vector transfected cells (Fig. 2C). Similarly, the total HBV load was decreased in BANF1-overexpressing Hep G2.2.15 cells (Fig. 2D). These results suggest that BANF1 possess antiviral activity against HBV.

### The expression of HBV-encoded proteins and total HBV viral load is downregulated significantly by BANF1-wild type compared to unphosphorylatable BANF1 mutants.

Previously it was shown that when thr-2, thr-3, and ser-4 residues were replaced with alanine, BANF1 was no longer phosphorylated, and hence, BANF1 could bind with dsDNA strongly and further decrease the total herpes simplex viral load (14, 15). Literature suggests that wild type (WT)-BANF1 can be phosphorylated and upon phosphorylation, BANF1 loses its ability to interact with DNA. Also, phosphorylation/dephosphorylation of BANF1 regulates the subcellular localization of BANF1. Unphosphorylatable BANF1 mutants are primarily expressed in the nucleus, while phosphorylated form of BANF1 translocates to cytoplasm (14). Hence, three BANF1 mutants, namely, Mutant1 (M1) (TTS**–A**TS), Mutant2 (M2) (TTS**–AA**S), and Mutant3 (M3) (TTS**–AAA**) were created by site directed mutagenesis (SDM). WT-BANF1 contains phosphorylation site, and upon phosphorylation loses its ability to bind dsDNA and translocates to cytoplasm, while unphosphorylated BANF1 mutants were hypothesized to remain bound with HBV cccDNA for longer duration and thus inhibit replication and transcription.

**FIG 3:**
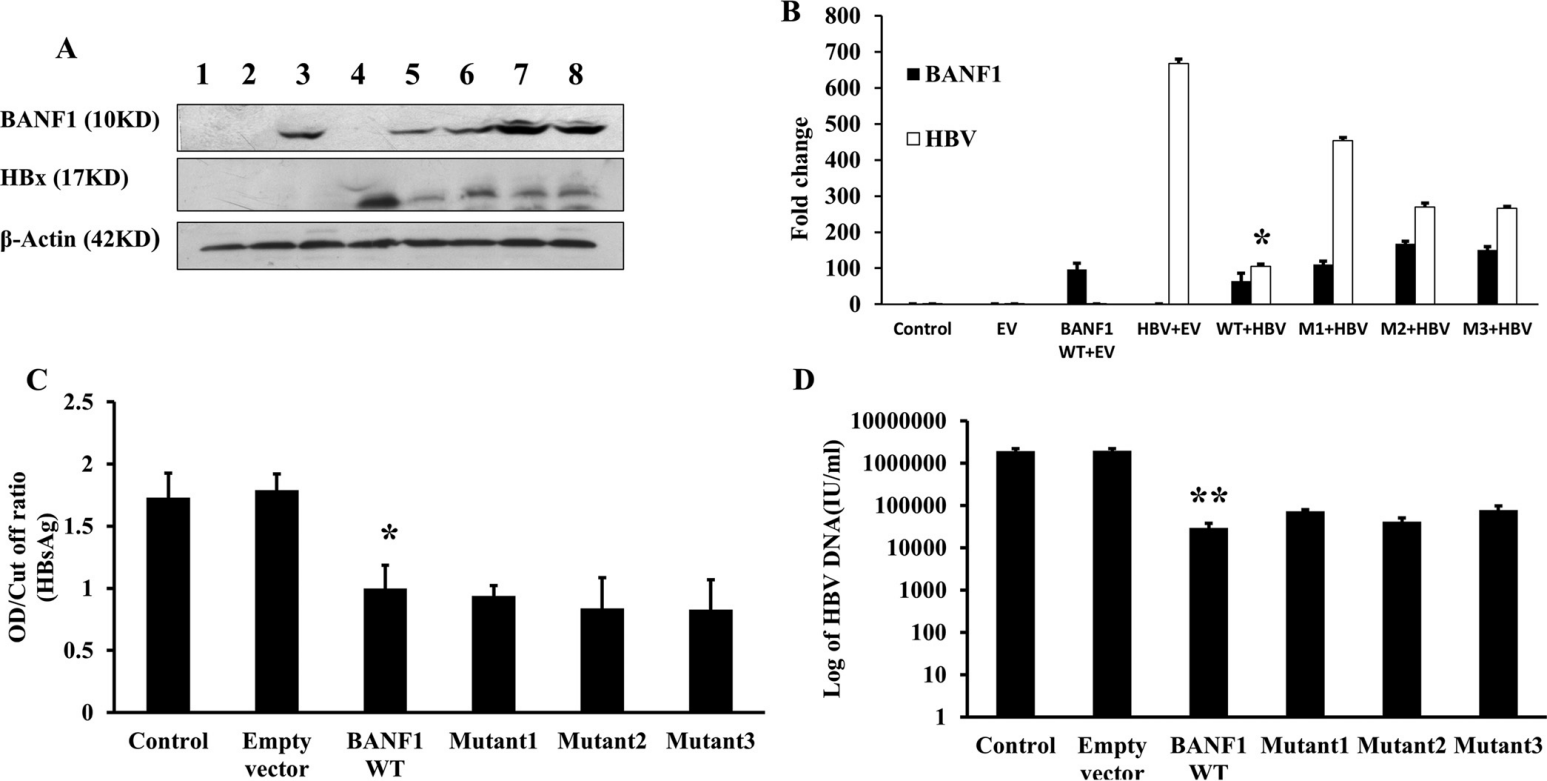
Mutation at the N-terminus of BANF1 modulates the expression of HBx, HBsAg and HBV replication. Huh-7 cells were transfected either alone with pHBV_1.3_ plasmid or cotransfected with BANF1 (WT), M1, M2, or M3 plasmid. After 48 h cells were harvested for total cellular protein isolation and Western blots were performed. A representative picture is shown. Lane 1, control; lane 2, empty vector transfected cells; lane 3, BANF1 transfected cells; lane 4, pHBV_1.3_ transfected cells; lane 5, BANF1(WT) and pHBV_1.3_ cotransfected cells; lane 6, M1 and pHBV_1.3_ cotransfected cells; lane 7, M2 and pHBV_1.3_ cotransfected cells; lane 8, M3 and pHBV_1.3_ cotransfected cells (A). The band intensities were quantified using Image J software and presented as fold change (HBx/β-actin, BANF1/β-actin) (*n* = 3; *, *P* < 0.05) (B). Hep G2.2.15 cells were transfected with BANF1 (WT), M1, M2, and M3 plasmid and after 48 h media was collected for HBsAg analysis by Enzyme Linked Fluorescent Assay (ELFA) (*n* = 3; *, *P* < 0.05) (C) and cells were harvested for HBV DNA isolation and quantification by qPCR (*n* = 3; ***, *P* < 0.001) (D).

BANF1 WT plasmid and BANF1 mutants (M1, M2, M3) were cotransfected with pHBV_1.3_ in Huh-7 cells. Western blot analysis revealed that in BANF1 WT and pHBV_1.3_-cotransfected cells there was a 6.4-fold decrease in the expression of HBx (Fig. 3A and B). In the cells cotransfected with pHBV_1.3_ and M1 or M2 or M3, there was a 1.5-fold, 2.5-fold, and 2.5-fold decrease in the expression of HBx, respectively (Fig. 3A and B). HBx and BANF1 band intensity quantification further confirmed that HBx expression was downregulated more significantly in BANF1-WT and HBx cotransfected samples compared to BANF1 mutants (M1, M2, M3) and HBx cotransfected samples (Fig. 3B). To further validate these results, HBV expressing Hep G2.2.15 cells were transfected with BANF1 WT, M1, M2, or M3 plasmid. After 48 h, spent media was collected for HBsAg analysis by ELISA and cells were collected to determine the total viral load by RT-PCR. Result showed that decrease in HBsAg levels due to BANF1 WT and BANF1 mutants were similar (Fig. 3C). Decrease in total HBV viral load was also similar in all the transfected cells (WT and mutants) and BANF1 mutants did not modulate the HBV expression further as hypothesized (Fig. 3D). These results indicate that BANF1-mediated HBV inhibition does not involve phosphorylation/dephosphorylation of BANF1 because unphosphorylatable BANF1 mutants, which are predominantly present in nucleus and also remain bound to DNA for a longer period, could not induce more intense inhibition of HBV compared to WT-BANF1.

### HBx upregulates miR-203 expression in hepatic cells.

**FIG 4:**
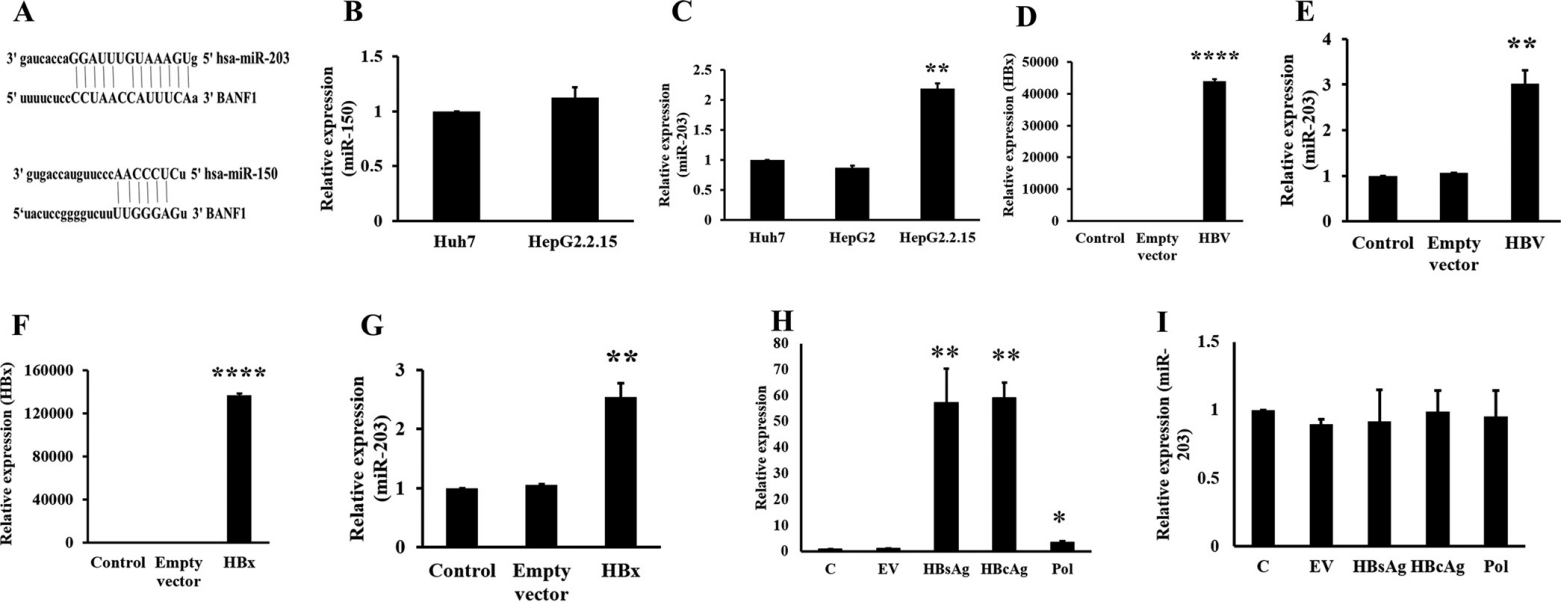
HBx upregulates miR-203 expression in hepatic cells. BANF1 targeting miRNAs (miR-150, miR-203) were forecasted using miRNA target prediction tools (TargetScan and miRBase) (A). Total RNA was isolated from Huh-7, Hep G2 and Hep G2.2.15 cells followed by RT-PCR to compare the expression of miR-150 (B) and miR-203 (*n* = 3; **, *P* < 0.01) (C). Huh-7 cells were transfected with pHBV_1.3_ plasmid and the cells were collected for the isolation of RNA, followed by RT-PCR for HBx (D) or miR-203 (E) expression (*n* = 3; ***, *P* < 0.001; **, *P* < 0.01). Huh-7 cells were transfected with HBsAg, HBcAg and HBV-pol plasmid, total RNA was isolated and RT-PCR was performed for HBsAg, HBcAg and HBV-pol (*n* = 3; *, *P* < 0.05; **, *P* < 0.01) (H) and miR-203 (I). After 48 h, RNA was isolated and RT-PCR was performed for the expression of HBx (F) or miR-203 (G) (*n* = 3; ***, *P* < 0.001; **, *P* < 0.01).

HBV infection deregulates various miRNAs, and these miRNAs in turn help in successful HBV infection establishment (16). To test whether miRNAs target BANF1 expression, miRNA databases (TargetScan and miRBase) were searched and it was found that both miR-203 and miR-150 are possible regulators (Fig. 4A). Total RNA was isolated from Huh-7, Hep G2 cells, and Hep G2.2.15 cells and RT-PCR was performed to compare the expression of miR-150 and miR-203. There was no significant change in the expression of miR-150 (Fig. 4B). MiR-203 expression was upregulated by 2-fold in Hep G2.2.15 cells compared to Huh-7 cells and Hep G2 cells (Fig. 4C). Next, Huh-7 cells were transfected with pHBV_1.3_ and total RNA was isolated and RT-PCR was performed for HBx and miR-203 expression. There was a significant expression of HBx (Fig. 4D) and miR-203 expression (Fig. 4E) in HBV transfected cells. To study whether the increased expression of miR-203 is due to HBx protein, Huh-7 cells were transfected with HBx plasmid and the total RNA was isolated. The RT-PCR results showed that there was a significant increase in the expression of HBx (Fig. 4F) and miR-203 (Fig. 4G) in HBx-transfected cells. To further validate these results, Huh7 cells were transfected with HBsAg, HBcAg and HBV polymerase (three additional proteins encoded by HBV apart from HBx) expressing plasmids and performed RT-PCR to evaluate the change in expression of miR-203. Results showed that HBsAg, HBcAg, and HBV-pol were upregulated (Fig. 4H) but there was no change in expression of miR-203 in HBsAg, HBcAg, and HBV pol transfected cells (Fig. 4I). These results suggest that HBV accessory protein HBx upregulates the expression of miR-203 in HBV infected cells, at least in part.

### miR-203 targets the BANF1 expression and increases the HBV viral titer.

**FIG 5:**
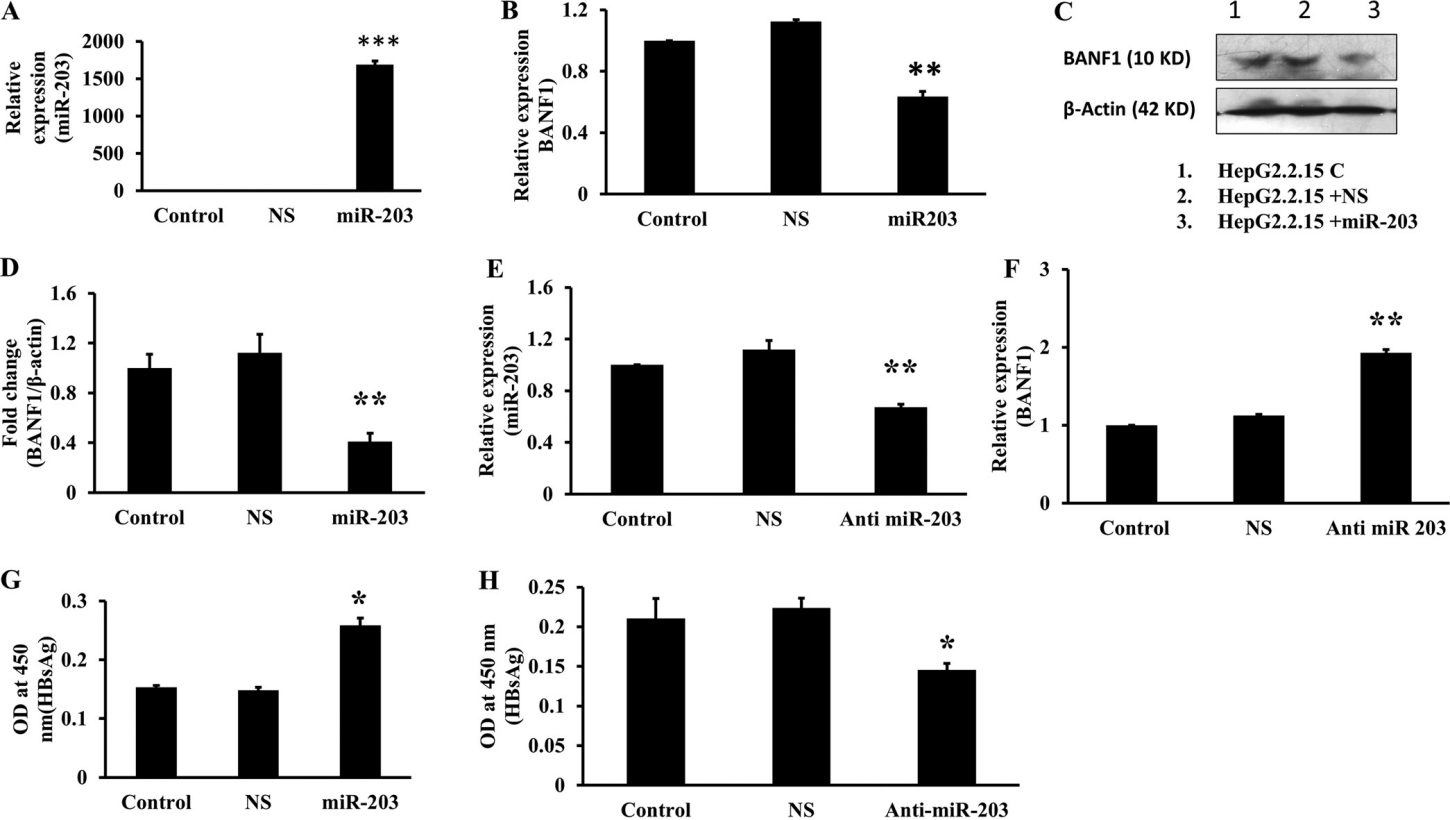
miR-203 downregulates BANF1 expression in HBV expressing cells. Hep G2.2.15 cells were transfected with miR-203 pre-miR oligos and after 48 h total cellular protein was isolated, and RNA was isolated after 72 h. RT-PCR was performed for miR-203 expression (A) and BANF1 expression (B) (*n* = 3; ***, *P* < 0.001; **, *P* < 0.01). The total cellular protein was run on SDS-PAGE gels and Western blots were performed and a representative picture is shown. Lane 1, control; lane 2, NS transfected cells; and lane 3, miR-203 transfected cells (C). The band intensities were quantified using Image J software and presented as fold change (BANF1/b-actin) (*n* = 3; **, *P* < 0.01) (D). Hep G2.2.15 cells were transfected with anti-miR-203 oligos and after 72 h RNA was isolated followed by RT-PCR for miR-203 expression (E) and BANF1 expression (F) (*n* = 3; **, *P* < 0.01; **, *P* < 0.01). Hep G2.2.15 cells were transfected with either miR-203 oligos and anti-miR-203 oligos, respectively. After 48 h spent media was collected for HBsAg analysis (G), (H) (*n* = 3; *, *P* < 0.05; *, *P* < 0.05).

Hep G2.2.15 cells were transfected with miR-203 pre-miR oligos and the expression of miR-203 and BANF1 were determined. The RT-PCR results showed that there was a significant increase in the miR-203 levels (Fig. 5A) and the BANF1 expression was significantly downregulated (Fig. 5B) in miR-203 overexpressing cells. Next, BANF1 protein expression was determined using Western blots in these cells. The results showed that there was a significant decrease of BANF1 in miR-203 transfected Hep G2.2.15 cells (Fig. 5C and D). To confirm these findings, anti-miR-203 was transfected in Hep G2.2.15 cells and the expression of miR-203 and BANF1 were determined using RT-PCR. The results showed that there was a 40% decrease in the expression of miR-203 (Fig. 5E), while the expression of BANF1 was upregulated (Fig. 5F) in anti-miR-203 transfected cells. Furthermore, Hep G2.2.15 cells were transfected with miR-203, and anti-miR-203, and after 48 h, spent media was collected for HBsAg analysis by ELISA. HBV viral titer was determined by estimating HBsAg level. ELISA results showed that HBsAg expression was upregulated in miR-203 transfected Hep G2.2.15 cells significantly (Fig. 5G) while HBsAg levels were significantly declined in ant-miR-203 transfected Hep G2.2.15 cells (Fig. 5H). These results thus established the link between miR-203, BANF1, and HBV interplay and suggested that HBx mediated miR-203 upregulation in HBV infected cells downregulates BANF1 expression and increases HBV titer.

## DISCUSSION

Chronic HBV infection is one of the leading causes for hepatocellular carcinoma. Once HBV infect the hepatocytes, it regulates the expression of various host proteins and signaling pathways to its favor for survival and replication. Previously, it was shown that Farnesoid X receptor-α and Polo-like-kinase 1 act as proviral factors during HBV infection (17, 18), while Talin-1 and SMC5/6 were reported to act as host restriction factors (2, 3). Identification of such HBV interacting host proteins may provide a deeper insight into HBV life cycle, which might be used for future targeted therapy. Hence, in this study, we determined the role of BANF1 in HBV replication and the mechanisms by which HBV inhibit BANF1 expression.

The DNA binding protein BANF1 is highly conserved and there are six BANF1 homologous genes in mammals (19). BANF1 exits in cytoplasm as well as nucleus of the cell. These nuclear and cytoplasmic pools are dynamic in nature, but studies showed that they cannot replenish each other (20). BANF1 interacts with LEM domain proteins and plays an important role in the nuclear envelope assembly during the late phase of mitosis (21, 22). Apart from its role in mitosis, BANF1 is involved in other cellular processes like gene expression (19), developmental process (23), cell cycle progression (21), and DNA damage response in the nucleus (24). BANF1 can sense and bind to foreign DNAs in the cytoplasm. By virtue of this, BANF1 associates with retroviral DNA preintegration complexes and protects it from undergoing suicidal autointegration (25). The ability of BANF1 to interact with dsDNA in a sequence independent manner allows it to act as host defense against HSV1 and vaccinia virus (14, 26). To date, there is no data available to show the relationship between BANF1 and HBV replication, and the mechanism by which HBV inhibits BANF1 expression in hepatic cells.

First, the effect of HBV on BANF1 expression was studied. It was found that BANF1 expression was significantly low in Hep G2.2.15 cells (HBV stably expressing Hep G2 cells) compared to Hep G2 cells. The expression of BANF1 was also tested in another hepatic cell line, Huh-7 cells, which showed the BANF1 protein levels were similar to Hep G2 cells, and not significantly different. These results showed that different biological features of cells probably do not affect the BANF1 expression, rather the change is due to HBV infection. To confirm these findings, Huh-7 cells were transfected with HBV plasmid (pHBV_1.3_), HBx plasmid, HBsAg plasmid, HBcAg plasmid or HBV-pol expressing plasmid and found that HBV inhibited the intracellular expression of BANF1 via HBx, at least in part. These results showed that the HBV infection inhibited BANF1 expression irrespective of cell lines. Next, the role of BANF1 in modulating HBV life cycle was established. The overexpression of BANF1 in Hep G2.2.15 cells resulted in decreased expression of HBsAg and HBV DNA (viral load). When Huh-7 cells were cotransfected with BANF1 and HBV plasmid, the HBx protein levels were decreased. These findings correlated well with the previous findings where the authors showed that BANF1 inhibited both HSV1 and vaccinia virus in L929 cells (14, 26).

Poxviruses express a Ser/Thr protein kinase B1 which phosphorylates the N-terminal serine threonine residues of BANF1 and renders it incapable of binding with poxviral DNA (13). In the case of HSV-1, it was found that BANF1 mutants that cannot be phosphorylated, impaired viral DNA replication (14). BANF1 showed a well-documented antiviral activity against cytoplasmic and nuclear viruses, but still its role in HBV life cycle is not known. Because HBV belongs to hepadnaviridae family and replicates its DNA genome in the nucleus, the phosphorylation/dephosphorylation of BANF1 was hypothesized to be responsible for its anti-HBV activity. WT-BANF1 (Met-Thr-Thr-Ser) contains ser/thr amino acids at its N-terminus which can be phosphorylated either by cellular vaccinia related kinase or by B1 kinase in case of Pox virus infection (13, 15). BANF1 phosphorylation renders it incapable of interacting with DNA and it can no longer induce cross bridging and condensation (14). Previous reports suggest that phosphorylation/dephosphorylation of BANF1 regulates the subcellular localization of BANF1 (14). Because WT-BANF1 contains phosphorylation site and upon phosphorylation loses its ability to bind dsDNA and translocates to cytoplasm, it was hypothesized that mutants will be retained in nucleus only and will remain bound to HBV DNA genome for longer period of time, thus leading to more inhibition compared to WT-BANF1.

To determine whether mutation resulted in enhanced inhibition of HBV levels, three different BANF1 mutants (M1, M2, M3), M1 (TTS**–A**TS), M2 (TTS**–AA**S), and M3 (TTS**–AAA**) were created by SDM and sequenced. The sequence results confirmed the changes in the correct positions. Overexpression of these mutants showed that these unphosphorylatabale BANF1 mutants still inhibited the replication, thereby decreasing total viral load, HBx and HBsAg, but as hypothesized, the inhibition induced by BANF1 mutants was not more than WT BANF1. These results led us to conclude that BANF1-mediated HBV inhibition does not involve cross bridging and condensation of HBV genome as reported for HSV-1, and inhibitory mechanism still remains elusive.

Several studies have shown that HBx is indispensable for HBV replication. HBx acts directly on viral promoters and enhancers to promote viral replication (4). HBx indirectly contributes to viral replication by modulating cellular pathways to benefit HBV infection (2, 5). Previously, we also showed that HBx induced proliferation via miR-21 in hepatic cells (27). Thus, HBx is indispensable for successful HBV infection and promotes replication from extrachromosomal templates (28). Though other proteins of HBV might play a role in modulating BANF1 expression, in this manuscript the role of HBx in HBV pathogenesis was studied.

HBV infection dysregulates expression of various miRNAs which in turn help in HBV infection establishment (16). To study if HBx modulates BANF1 expression via miRNA expression, miRNA databases (TargetScan and miRBase) were searched and based on the literature both miR-203 and miR-150 were selected, because they might target the 3′-UTR of BANF1 expression. It was found that expression of miR-203 was significantly upregulated in Hep G2.2.15 cells, while there was no change in expression of miR-150. Further the expression of HBx induced the expression of miR-203 in HBV infected cells; however, there was no change observed in expression of miR-203 in HBsAg, HBcAg, and HBV-pol transfected cells. Previously, it was shown that HBx-mediated miR-203 is involved in inducing inflammation (29). HBV infection leads to hepatocellular carcinoma progression via HBV-induced immune imbalance (30). Previous studies have shown that the overexpression of miR-203 reduced HCC prevalence, and could inversely affect the HCC outcome (31). Based on the available data with the progression of HCC and the HBV-induced HCC, it might be possible that different mechanisms exist. Inhibition of miR-203 using anti-miR-203 upregulated BANF1 expression. To establish the link between miR-203, BANF1, and HBV interplay, Hep G2.2.15 cells were transfected with miR-203 or anti-miR-203. The results confirmed that miR-203 upregulated the viral titer, while anti-miR-203 inhibited the viral titer. In summary, for the first time we showed that BANF1 inhibited the expression of HBx, HBsAg, and HBV DNA (viral load) in HBV-infected cells, and HBV inhibited BANF1 expression, at least in part, via HBx-mediated upregulation of miR-203 in hepatic cells.

## MATERIALS AND METHODS

### Cell culture and transfection experiments.

Huh-7, Hep G2, and Hep G2.2.15 (stably HBV expressing Hep G2 cells) cells were maintained in Dulbecco’s modified Eagle’s medium (DMEM, HiMedia Laboratories, Mumbai, India) supplemented with 1 mg/mL penicillin-streptomycin antibiotics (Gibco, Thermo Fisher Scientific, MA, USA) and 10% fetal bovine serum (FBS; Invitrogen, Carlsbad, CA, USA). The cells were transfected after 24 h with BANF1 (WT, M1, M2, M3), pHBV_1.3_ (Hepatitis B virus 1.3-fold genome plasmid, a kind gift from ILBS, New Delhi, India) and HBx expressing plasmids (HBx is a protein encoded by HBV and has been shown to be required for HBV replication), HBV surface antigen expressing (HBsAg) plasmid, HBV core protein expressing plasmid (HBcAg), and HBV polymerase expressing plasmid (HBsAg plasmid, HBcAg plasmid and HBV pol plasmid were a kind gift from Dr. B Nayak, AIIMS, New Delhi, India) or empty vector (pcDNA3.1), using lipofectamine 2000 (Thermo Fisher Scientific) according to the manufacturer’s instructions. After 24 h of transfection, the spent media was collected for HBsAg analysis, and the cells were harvested for DNA, RNA, or protein isolation. For miRNA transfection experiments, Hep G2.2.15 cells were transfected with either miRNA-203 mimic, anti-miR-203 oligos (Sigma-Aldrich, St. Louis, USA) or NS-miRNA (Sigma-Aldrich) using SiPortNeoFx reagent (Invitrogen, Thermo Fisher Scientific). The media was replaced after 24 h of transfection and the cells were harvested after 48 h for protein and RNA isolation.

### Cloning and SDM of BANF1 mutants.

BANF1(WT) from Huh-7 cells was amplified by PCR by using the primers listed in Table 1. The PCR product was digested with restriction enzymes EcoRI (NEB, Ipswich, MA, USA) and BAMH1 (NEB) and cloned into pcDNA3.1(-) vector. Using the specific primers with one or two or three mutations (Table 1), mutants (M1, M2, M3) were generated by using Quick-change II XL Site Directed Mutagenesis kit (Agilent Technologies, Santa Clara, CA, USA). SDM PCR was run according to the manufacturer’s instructions and cloned into pcDNA3.1(-). All the cloned and SDM generated vectors were confirmed by sequencing.

**TABLE 1:** List of primers used for RT-PCR

Genes	Primer sequences
BANF1 WT FP	5′-CCGGAATTCATGACAACCTCCCAAAAGCACCG-3′
BANF1 WT RP	5′-CGCGGATCCCAAGAAGGCGTCGCACCACTC-3′
BANF1 M1 FP	5′-CCGGAATTCATGACAACC**G**CCCAAAAGCACCG-3′
BANF1 M1 RP	5′-CGCGGATCCCAAGAAGGCGTCGCACCACTC-3′
BANF1 M2 FP	5′-GATATCTGCAGAATTCATG**G**CA**G**CCTCCCAAAAGCACCGAG-3′
BANF1 M2 RP	5′-CTCGGTGCTTTTGGGAGGCTGCCATGAATTCTGCAGATATC-3′
BANF1 M3 FP	5′-GATATCTGCAGAATTCATG**G**CA**G**CC**G**CCCAAAAGCACCGAG-3′
BANF1 M3 RP	5′-CTCGGTGCTTTTGGGAGGCTGCCATGAATTCTGCAGATATC-3′
BANF1 RT FP	5′-AGGTAGAGAGACCCTTTGGTTAG-3′
BANF1 RT RP	5′-CTATCACTCCCTCTGTGACCTT-3′
HBsAg RT FP	5′-CACCTGTATTCCCATCCCATC-3′
HBsAg RT RP	5′-CCCTACGAACCACTGAACAAA-3′
HBcAg RT FP	5′-CAATGTCAACGACCGACCTT-3′
HBcAg RT RP	5′-GCCTACAGCCTCCTAGTACA-3′
HBV Pol FP	5′-AGGTCTGTGCCAAGTGTTTG-3′
HBV Pol RP	5′-TCCCGATAATGTTTGCTCCAG-3′
HBx RT FP	5′-ACCGACCTTGAGGCCTACTT-3′
HBx RT RP	5′-GCTTGGCAGAGGTGAAAAAG-3′
GAPDH RT FP	5′-ACCAGGTGGTCTCCTCTGAC-3′
GAPDH RT RP	5′-TGCTGTAGCCAAATTCGTTG-3′

### Western blotting.

The cells were harvested after 48 h of transfection using the mammalian protein extraction reagent (mPER; Thermo Fisher Scientific) supplemented with protease inhibitor cocktail (PIC, Thermo Fisher Scientific). Bicinchoninic acid (BCA) assay was performed for the estimation of protein concentration. Total cellular protein (40 μg per well) was run in the SDS-PAGE gels, transferred onto a PVDF membrane, blocked using bovine serum albumin fraction-V (HiMedia) and were incubated overnight with BANF1 (Abcam, Cambridge, UK), HBx (Santa Cruz Biotechnology, Dallas, TX, USA), or βactin (Santa Cruz). PVDF membranes were washed 6 × 5 min each and incubated with HRP-conjugated corresponding secondary antibodies and developed using ECL kit (Thermo Fisher Scientific) on photosensitive films.

### RNA isolation, cDNA synthesis, and RT-PCR.

The total RNA was isolated using TRIzol reagent as per the manufacturer’s instructions (Thermo Fisher Scientific; Cat. No. 15596026). The cDNA was synthesized from the total RNA using, Universal cDNA synthesis kit II (Exiqon, Vedbaek, Denmark) for miRNA expression analysis or TaKaRa cDNA synthesis kit for mRNA expression analysis. Primers for miRNA-203 (Exiqon), 5S rRNA (Exiqon), BANF1, HBx, HBsAg, HBcAg, HBV-pol and GAPDH (Integrated DNA Technologies, Skokie, IL, USA) (Table 1) were used for real-time PCR using SYBR green in a ViiA 7 real-time PCR system (Applied Biosystems, Thermo Fisher Scientific). Expression analysis of all the genes was done using 2^-ΔΔCt^ method as described previously (32).

### HBV DNA isolation and quantification.

HBV DNA isolation and quantification protocol was followed as described previously (33). Briefly, Hep G2.2.15 cells transfected with BANF1 were harvested after 48 h and cell pellet was lysed by addition of autoclaved milli Q water and TE saturated phenol. The tubes were incubated at 65°C for 2 h and then centrifuged at 12,000 rpm for 10 min followed by addition of 200 μL chloroform. The supernatant precipitation was carried out with 7.5 M ammonium acetate and 100% ethanol. DNA precipitate was washed with 70% ethanol, air dried for approximately 20 min and stored at −20°C. HBV DNA quantification was performed by qPCR using primers (Table 1) and a TaqMan probe (HBV1570-5′FAM CGGACCGTGTGCACTTCGCTT-BHQ) of X region of HBV. Standard curve was derived by using serial dilutions (2 to 8 log_10_) of HBV-DNA High control (Acrometrix, Thermo Fisher Scientific) which also served as a control. HBV DNA panel (Acrometrix, Thermo Fisher Scientific) compared with the reference (WHO HBV control) was used to standardize HBV DNA quantity. HBV DNA quantity was analyzed in international units per mL (IU/mL).

### Enzyme linked fluorescent assay.

Hepatitis B surface antigen (HBsAg) in cell culture supernatant was detected by enzyme linked fluorescent assay (ELFA) in automated benchtop immunoanalyzer VIDAS (BIOMÈRIEUX). HBsAg detection in 100 μL of cell culture supernatant was carried out by using VIDAS HBS kit as per manufacturer's protocol. HBsAg titer was derived from sample/cut off standard ratio using relative fluorescent value (RFV). Samples with ratio values < 0.13 were considered negative and with ratio values > 0.13 were considered positive and sample titer mentioned accordingly. The percentage inhibition was determined on the basis of sample: cut off RFV.

### ELISA.

Hepatitis B surface antigen (HBsAg) in cell culture supernatant was detected by enzyme linked immunosorbent assay (ELISA). HBsAg detection in 100 μL of cell culture supernatant was carried out by using Monolisa HBs Ag ULTRA (Bio-Rad) kit as per manufacturer's protocol. Briefly, 100 μL of cell culture supernatant along with positive- and negative-control samples (provided in kit) were loaded in triplicates into enzyme coated strips followed by addition of enzyme conjugate solution. The plate was incubated at 37°C for one and half hour. Strips were washed six times with provided washing solution. Chromogenic substrate was added in dark and incubated for half an hour followed by OD measurement at 450 nm in enzyme plate reader (Bio-Rad). The cutoff value was calculated using the following formula. Cutoff value = Mean absorbance of negative control + 0.1. The samples with OD more than cutoff value were considered positive.

### Statistical analysis.

All the experiments shown were performed at least three times (*n* = 3) and in duplicates. The statistical significance (*P* value) was calculated using unpaired Student's *t* test while comparing between two groups. For the Western blot analyses, a blot representative of one of the three experiments is shown. Data were considered significant if *P* < 0.05.
